# Role of the Qinghai-Tibetan Plateau uplift in the Northern Hemisphere disjunction: evidence from two herbaceous genera of Rubiaceae

**DOI:** 10.1038/s41598-017-13543-5

**Published:** 2017-10-17

**Authors:** Tao Deng, Jian-Wen Zhang, Ying Meng, Sergei Volis, Hang Sun, Ze-Long Nie

**Affiliations:** 1 Key Laboratory for Plant Diversity and Biogeography of East Asia, Kunming Institute of Botany, Chinese Academy of Sciences, Kunming, Yunnan 650201 China; 2 Key Laboratory of Plant Resources Conservation and Utilization, College of Biology and Environmental Sciences, Jishou University, Jishou, Hunan 416000 China

## Abstract

To assess the role of the Qinghai-Tibetan Plateau uplift in shaping the intercontinental disjunction in Northern Hemisphere, we analyzed the origin and diversification within a geological timeframe for two relict herbaceous genera, *Theligonum* and *Kelloggia* (Rubiaceae). Phylogenetic relationships within and between *Theligonum* and *Kelloggia* as well as their relatives were inferred using five chloroplast markers with parsimony, Bayesian and maximum-likelihood approaches. Migration routes and evolution of these taxa were reconstructed using Bayesian relaxed molecular clock and ancestral area reconstruction. Our results suggest the monophyly of each *Theligonum* and *Kelloggia*. Eastern Asian and North American species of *Kelloggia* diverged at ca.18.52 Mya and the Mediterranean species of *Theligonum* diverged from eastern Asian taxa at ca.13.73 Mya. Both *Kelloggia* and *Theligonum* are Tethyan flora relicts, and their ancestors might have been occurred in warm tropical to subtropical environments along the Tethys coast. The Qinghai-Tibetan Plateau separated the eastern and western Tethyan area may contribute significantly to the disjunct distributions of *Theligonum*, and the North Atlantic migration appears to be the most likely pathway of expansion of *Kelloggia* to North America. Our results highlight the importance role of the QTP uplift together with corresponding geological and climatic events in shaping biodiversity and biogeographic distribution in the Northern Hemisphere.

## Introduction

Clarifying the major factors underlying intercontinental disjunct distributions in the Northern Hemisphere has long been regarded as one of the central problems of plant biogeography^1^. Both the North Atlantic land bridge (NALB) and the Beringian land bridge (BLB) were available for plant migration during the Cenozoic^2^, but their availability for movement of particular clades of plants and animals fluctuated with changes in physical connectivity and climate^2–4^. Despite significant progresses have been achieved in understanding patterns of disjunction around the Northern Hemisphere, many important questions need to be clarified, such as the vicariance role of the Qinghai-Tibetan Plateau (QTP) uplift and subsequent effects on plant distribution in the Northern Hemisphere.

Since the Cretaceous, one of the most remarkable geological changes in Eurasia is the uplift of the QTP that resulted from the collision of the Indian Plate with Eurasian Plate in early Cenozoic^5–7^. These collision and uplift also closed the Tethys Sea followed with the permanent closure of the Turgai Seaway and contributed significantly to continentalization in Europe in the middle to late Cenozoic^8^. All these events opened up new corridors for biotic exchange and created various types of new habitats, which produced great effects on climate and biodiversity in the Northern Hemisphere^9,10^. The QTP is finally shaped as the highest and one of the most extensive plateaus in the world and one of the biodiversity hotspot in the north temperate region which harbors more than 12,000 species of vascular plants in 1500 genera^11,12^. Studies of plant diversification within the QTP suggested multiple mechanisms of adaptive radiation involved^13^. However, the impact of the QTP uplift on the biogeographic pattern in the Northern Hemisphere received relatively less attention, especially for its effects on the intercontinental disjunction in plants.

Climate cooling began in the Middle Eocene (but see Prothero, 1994^14^ for an exception) and subsequent aridification in the Miocene–Pliocene are commonly accepted as the main causes of disjunctions between floristic elements of eastern Asia and western Eurasia from the once widespread Cenozoic flora^1,2,4,15–18^. The important contribution of the uplift of the QTP and downstream influences to these processes has been recognized^19–22^. By changing the regional climate and creating a physical barrier to flora exchange, the uplift had various impacts on the biodiversity of the QTP and adjacent areas including extinctions, floristic reorganizations and adaptive radiation^23–25^. Particularly, the QTP uplift might play an important role on shaping the well-known Madrean-Tethyan disjunction in the Northern Hemisphere.

The Madrean-Tethyan disjunction was suggested by Raven^26^ and Axelrod^27,28^ and reviewed by Liston^29^ and Wen and Ickert-Bond^30^ from phylogenetic perspectives. It was hypothesized a nearly continuous trans–Atlantic belt of Madrean-Tethyan dry and broadleaf evergreen sclerophyllous vegetation that stretched from western North America to Central Asia in the early Cenozoic at low latitudes^28^. The QTP is located on the east end of this belt. With continuous uplift of QTP and the spread of cool and dry climates after middle to late Eocene, broadleaved evergreen taxa were replaced by more mesic elements occupied in more restricted subhumid or dry forests across the Madrean-Tethyan regions^31^. The disjunct pattern is ancient and results of recent biogeographic studies mostly favor NALB migration route between the Old and the New World, but this hypothesis still needs to be verified with additional analyses^30^. Moreover, others have argued for the origin of this disjunct pattern via BLB or resulting from long-distance dispersal^1,30,32^.

Understanding of the plateau uplift history has been advanced by the application of paleontology and stable isotopes to studies of the Tibetan Plateau^10^. It is generally accepted that the QTP uplifted multiple times at different scales and the Himalayas reached their current elevation in the middle-late Miocene, but the uplift histories of the different terranes that comprise this plateau currently remain unclear^9,10^. Although the effects of QTP uplift, continentalization in Europe and aridification in Central Asia in Cenozoic in producing many biogeographic disjunctions are well recognized^19,22,33–36^, very few plant taxa has been evolved to elaborate the detailed date and process of biotic evolution during or after this uplift.

*Theligonum* L. and *Kelloggia* Torrey ex Bentham, two small genera from the coffee family (Rubiaceae), are excellently suited taxa to infer the role of the QTP uplift in producing disjunct distributions in the Northern Hemisphere. Both genera have a disjunct distribution along the Madrean–Tethyan belt and across the two sides of the QTP. *Theligonum* is a prostrate herbaceous genus occupying humid microenvironment and comprising four species: three are found at high elevations of 2500–2800 m in temperate regions of eastern Asia^37,38^; and one occurs at low altitude around 600–900 m in Macaronesia, the Mediterranean and the Near East^39^. *Kelloggia* includes only two species: *K*. *chinensis* Franch., that occurs in alpine meadows or forest clearances at above 3000 m on the eastern Tibetan Plateau and *K*. *galioides* Torrey that grows in open places of coniferous forests (1100–3000 m) in the western North America^40–43^.

Recent molecular studies identify *Theligonum* and *Kelloggia* as the closest relatives of tribe Rubieae^40,44–46^, which is centered in temperate regions and is one of the largest herbaceous tribes of Rubiaceae^46–48^. Although the sister relationship of *Theligonum* with *Kelloggia* plus Rubieae has been reported^40^, the biogeography and evolutionary history of the two genera have not been fully understood. *Theligonum* is an isolated genus of the Cenozoic evergreen forest^33^ and its monophyletic status has never been tested with sampling from eastern Asia. The divergence time for *Kelloggia* between eastern Asia and western North America was dated back to 5.42 ± 2.32 Mya based on only *rbc*L sequence^40^. According to this result, Nie *et al*.^40^ suggested the intercontinental disjunction in *Kelloggia* was evolved via long-distance dispersal from Asia into western North America. It did not support the Madrean-Tethyan hypothesis. However, as the authors pointed out this conclusion is based on a single gene region and limited taxa representation necessitating further analysis. Given the distribution of the eastern Asian species *K*. *chinensis* (about 3000 m, western edge of eastern Asia close to Central Asia) and the occurrence of many close relatives of *Kelloggia* (such as *Putoria* Pers. and *Plocama* Aiton in the tribe Paederieae and *Galium*) in the Mediterranean^49^, the genus origin and evolution seem to be closely related to the ancient Madrean-Tethyan region and the QTP uplift.

A more representative sampling of *Theligonum* and *Kelloggia* may provide higher phylogenetic resolution and improved estimation of divergence times for these taxa as well as crucial insights into the effect of the QTP uplift on plant evolution in the Northern Hemisphere. Thus, the purpose of this study was to estimate divergence times and ancestral areas for *Kelloggia–Theligonum* and their close relatives in order to reconstruct the biogeographical history of both *Kelloggia* and *Theligonum*. Primarily, we aimed to determine whether the current distribution of these two genera was affected by the uplift of QTP, migration along the land bridges during the late Oligocene to Miocene, long-distance dispersal, or a combination of these. As similar distribution patterns characterize other temperate plant lineages of Northern Hemisphere, such as *Eremurus* (Asphodelaceae), *Parapteropyrum* (Polygonaceae), *Paliurus* (Rhamnaceae) and *Colutea* (Fabaceae), we suppose that our findings on *Kelloggia–Theligonum* evolution could have broad applications. Even more generally, we hope that our analyses will encourage increased attention to the effect of the QTP uplift on plant evolution in the Northern Hemisphere.

## Results

### Phylogenetic analyses

The combined five–marker (*rbc*L, *rps*16, *trn*T–F, *atp*B–*rbc*L and *psb*A–*trn*H) data matrix consisted of 5393 nucleotides. In the combined MP analyses, 1175 characters were variable, 633 of which were potentially parsimony–informative. The MP analyses resulted in > 10,000 equally MPTs with a length of 635 steps, a consistency index of 0.83, a retention index of 0.92 and a rescaled consistency index of 0.76. For the Bayesian analysis, all partitions had a best-fit model of GTR + G, with the exception of *rbc*L and *atp*B-*rbc*L, for which it was TVM + I + G and TVM + I, respectively.

**Figure 1:**
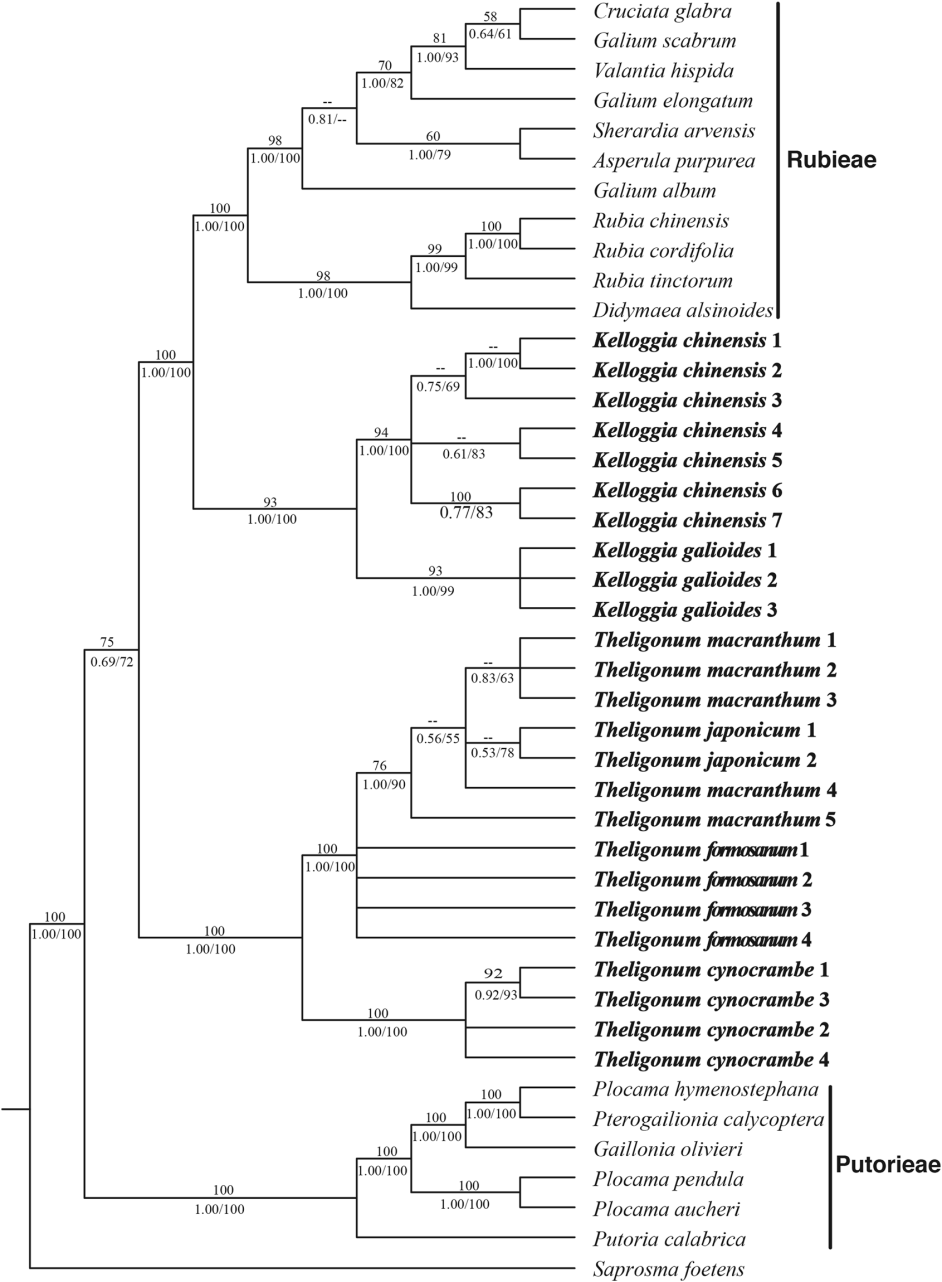
The Bayesian consensus tree of *Theligonum* and *Kelloggia* and related taxa from Rubieae and Putorieae based on five plastid sequences (*rbc*L, *rps*16, *trn*T–F, *atp*B–*rbc*L and *psb*A–*trn*H). The Bayesian posterior probabilities are shown above the branches and the MP/ML bootstrap values below.

The topologies from the maximum parsimony, Bayesian inference and maximum likelihood analyses were congruent, but varied in the level of support for some nodes (Fig. 1). Within *Kelloggia*, the sister position of *K*. *chinensis* and *K*. *galioides* was well supported (BP = 93; PP = 1.00; BS = 100). *Theligonum* was found to be monophyletic (BP = 100; PP = 1.00; BS = 100), with a basal dichotomy into two major clades (Mediterranean, i.e. *T*. *cynocrambe*, and the eastern Asian clade), each with high support (BP = 100; PP = 1.00; BS = 100). In the eastern Asian clade, the Taiwan endemic *T*. *formosamum* was sister to the other two species. The latter species, *T*. *macranthum* from central China and *T*. *japonicum* from eastern China and Japan formed a distinct group (Fig. 1).

### Divergence time analyses

**Figure 2:**
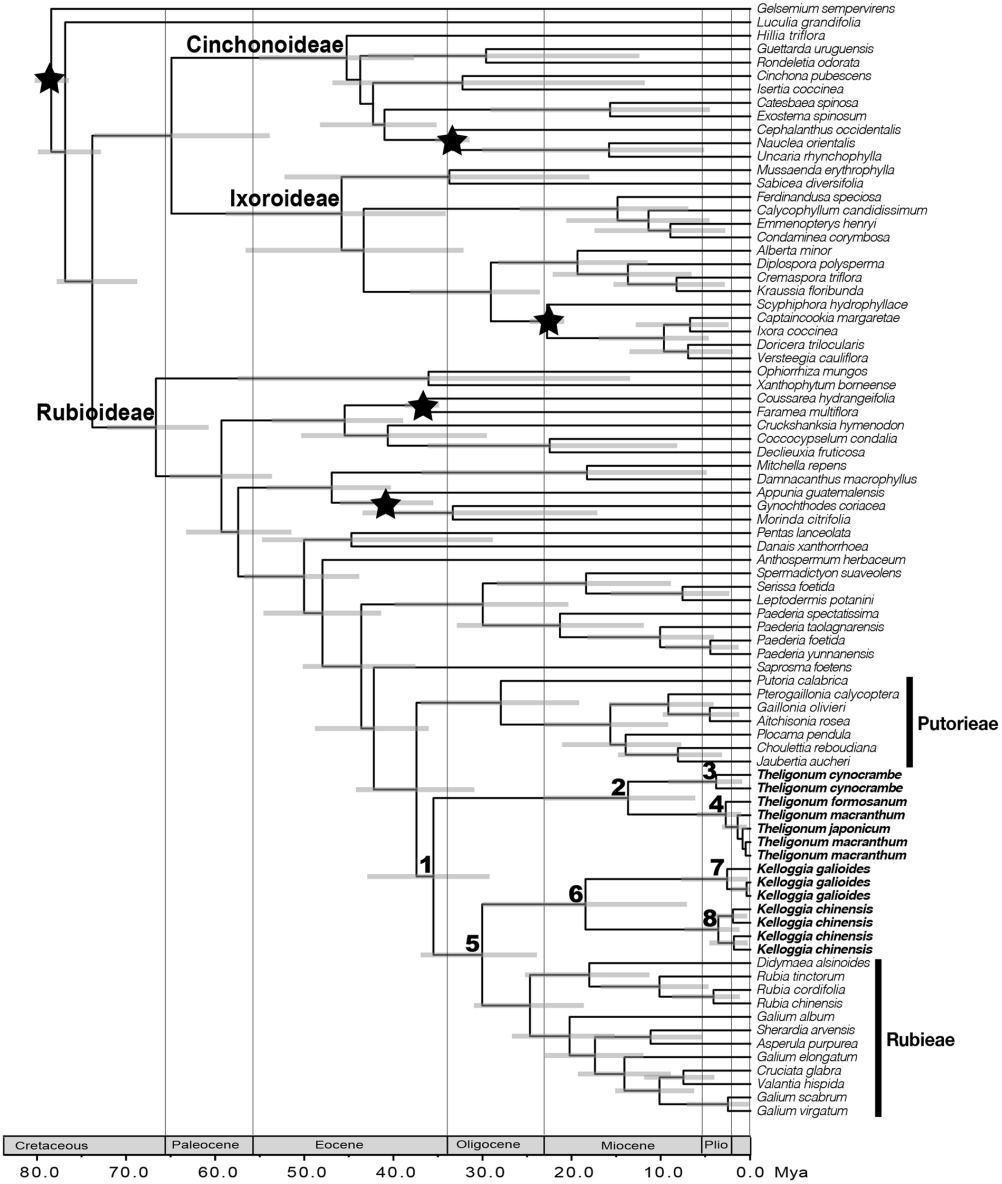
Chronogram of *Theligonum* and *Kelloggia* together with representatives from Rubiaceae inferred from BEAST. Grey bars represent the 95% highest posterior density intervals for node ages. Stars are calibration points; 1–8 indicate nodes with interests (see Table 1 for details).

The BEAST analysis generated a well–resolved tree for *Theligonum* and *Kelloggia*, the topology of which is consistent with the topologies from the MP and Bayesian analyses (Fig. 2). The uncorrelated–rates relaxed molecular clock suggested an origin of the *Theligonum* stem lineage in the late Eocene (35.57 million years ago (Mya); 95% HPD: 29.27–42.99 Mya; node 1 in Fig. 2). Within *Theligonum*, the split between the eastern Asian clade and the Mediterranean clade is dated at 13.73 Mya (95% HPD: 6.19–23.24 Mya; node 2 in Fig. 2). The age of the crown group of the eastern Asian subclade was estimated at 2.77 Mya (95% HPD: 1.03–6.01 Mya; node 4 in Tao Deng and Jian-Wen Zhang contributed equally to the work.), while the age of the crown group of the Mediterranean subclade was inferred as 3.86 Mya (95% HPD: 0.94–9.23 Mya; node 3 in Fig. 2).

The uncorrelated–rates relaxed molecular clock suggested an origin of the *Kelloggia* stem lineage in the early Oligocene (30.1 Mya; 95% HPD: 23.96–37.01 Mya; node 5 in Fig. 2). The spilt between the eastern Asian and the North American species was estimated at 18.52 Mya (95% HPD: 7.13–30.07 Mya; node 6 in Fig. 2).

### Ancestral area reconstruction

**Figure 3:**
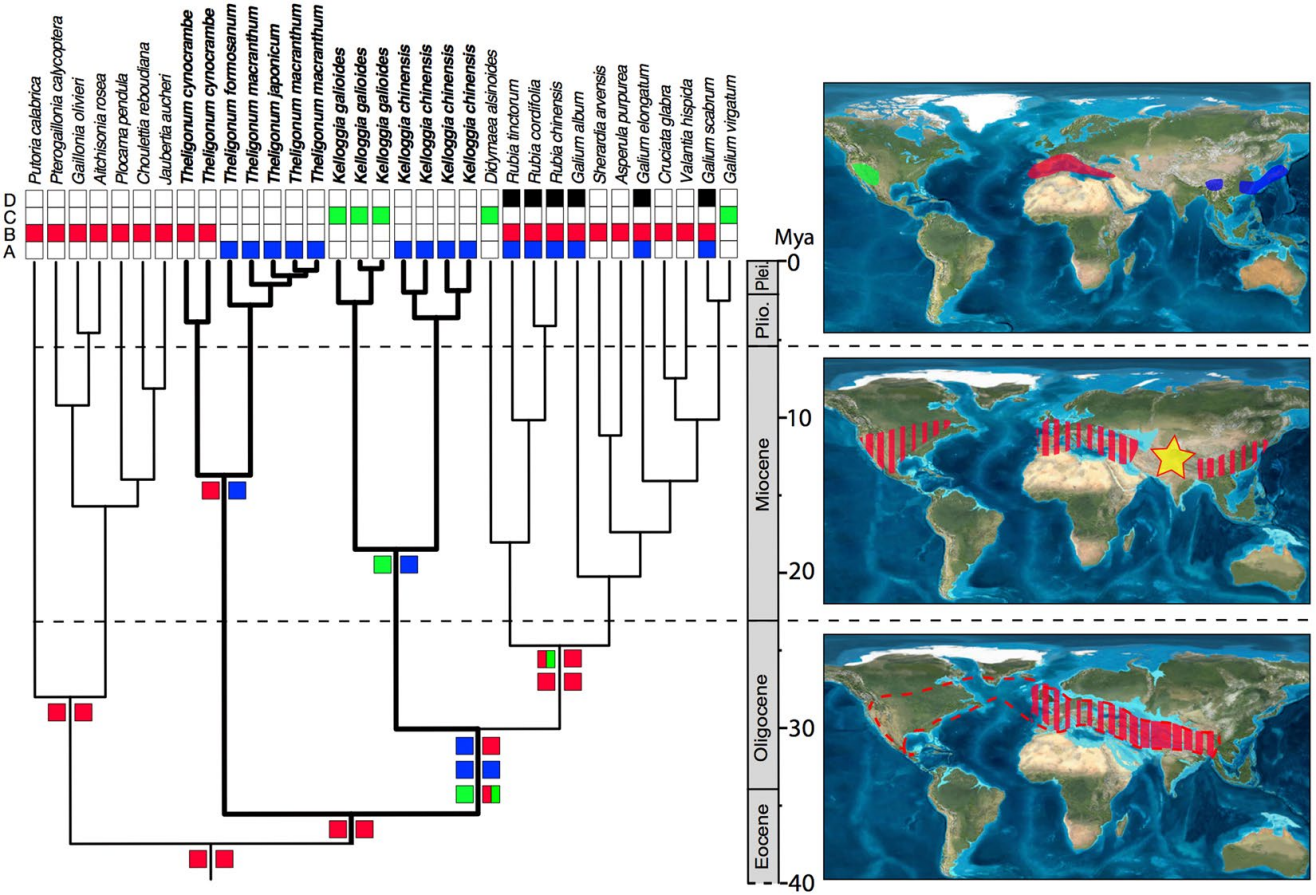
Ancestral area reconstruction of *Theligonum* and *Kelloggia* by Lagrange (left) with biogeographic scenario showing on the maps (right). A = eastern Asia; B = the Mediterranean (including western Asia and north Africa); C = North America; D = Africa, excluding north Africa. The uplift of QTP is indicated by a yellow star. Maps were generated using the software ArcGIS 9.3 (http://webhelp.esri.com/arcgisdesktop/9.3/index.cfm?TopicName=welcome).

Ancestral area reconstructions are illustrated in Fig. 3, and relative and marginal probability values of some important nodes are summarized in Table 1. Lagrange and S–DIVA analyses yielded highly similar results (differences are indicated in Table 1). Our analyses suggested western Eurasia as the ancestral area for *Theligonum*–*Kelloggia*–Rubieae clade (Fig. 3, Table 1; Mediterranean or eastern Asia in the S–DIVA analyses). Reconstruction for the *Theligonum* crown group indicated eastern Asia and/or western Eurasia as the most likely ancestral area. The genus splits into two lineages: one (*T*. *cynocrambe*) occurs in the western Eurasia and the other consisting of the rest of *Theligonum* was inferred as an ancestral area in eastern Asia. For *Kelloggia*, the optimizations for the stem and crown nodes indicated wide ancestral areas in eastern Asia, western North America and/or western Eurasia, and a vicariance event may have caused the disjunction between the eastern Asian and the North American clades (Fig. 3).

**Table 1:** Lineage divergence times and range inheritance scenarios for nodes of interest (Fig. 2) as estimated from BEAST, Lagrange, S-DIVA, respectively.

Node	Description	Mean age (95% HPD) (Mya)	LAGRANGE	S–DIVA
1	*Theligonum* and its closest relative *Kelloggia*–Rubieae	35.57 (29.27, 42.99)	B|B (0.37)	A (49.08) B (50.92)
2	Mediterranean and eastern Asian *Theligonum*	13.37 (6.19, 23.24)	B|A (0.90)	AB (100)
3	Crown of *Theligonum cynocrambe*	3.86 (0.94, 9.23)	B|B (0.99)	B (100)
4	Crown of EA *Theligonum*	2.77 (1.03, 6.01)	A|A (1)	A (100)
5	*Kelloggia* and its closest relative Rubieae	30.1 (23.96, 37.01)	C|BC (0.21) A|A (0.19) A|B (0.15)	BC (33.69) A (32.62) AB (33.51)
6	eastern Asian and North American *Kelloggia*	18.52 (7.13, 30.07)	C|A (0.78)	AC (100)
7	Crown of *Kelloggia galioides*	2.62 (0.31, 7.75)	C|C (0.98)	C (100)
8	Crown of *Kelloggia chinensis*	3.61 (1.23, 7.39)	A|A (0.99)	A (100)

## Discussion

### Tethyan origin of *Theligonum* and *Kelloggia*

Our results provide strong molecular phylogenetic support to the monophyly of *Theligonum*, which is consistent with many unique morphological features that distinguish the genus in Rubiaceae^38,39,50,51^. In our gene tree, *Theligonum* is sister to a clade including *Kelloggia* and Rubieae with moderate support, which is in agreement with other molecular phylogenetical^40,45,51^ and morphological studies^52^. Our results also strongly support the previously reported monophyly of *Kelloggia* and its sister relationship with the Rubieae clade^40,46^. The Putorieae was found to be monophyletic and sister to the *Theligonum*–*Kelloggia–*Rubieae group, as previously reported by Backlund *et al*.^45^. Putorieae are mostly shrubs or shrublets^45^ while Rubieae are predominantly herbaceous species, thus the detected phylogenetic position of the exclusively herbaceous *Theligonum* and *Kelloggia* indicate their key position in the evolutionary transition from the woody to herbaceous habit in Rubiaceae.

Our dating analysis suggests that the ancestor of *Theligonum* has arisen in late Eocene (35.57 Mya, node 1, Fig. 2), and *Kelloggia* separated from Rubieae in early Oligocene (30.2 Mya, node 5, Fig. 2). Many close relatives of *Theligonum* and *Kelloggia* are distributed in western Eurasia (e.g. *Plocama* in the tribe Putorieae), and Rubieae also has many taxa centered in Madrean–Tethyan region (such as *Galium* and *Rubia*) and a rich endemism in the Mediterranean region and Europe^40,49,53^. According to our ancestral area reconstruction and dating analysis, the two genera together with Putorieae and Rubieae originated in the Eocene to Oligocene, apparently along the Tethyan coast (Fig. 3). During the Eocene to Oligocene, the Madrean–Tethyan flora represented by sclerophyllous plants adapted to subhumid climate, inhabited lower–middle latitudes forming a belt along the shores of the Tethyan region and extending even to North America^54^. From the start of Oligocene, once widespread evergreen and woody flora moved south in response to climatic cooling, while many deciduous and herbaceous taxa appeared^18,54^. Furthermore, the Tethys coast was also considered as a refugium for warm adapted species during global cooling beginning in the Middle Eocene^14^. The Mediterranean waters or other factors probably maintained that region as suitable and relatively stable for warm-adapted species for some time while climate cooled more drastically elsewhere in the Northern Hemisphere^14^. Extant species of the two studied genera living in temperate zone near the southern part of ancient Tethyan region in humid or subhumid habitats can be the relict elements of the Palaeo-Tethyan flora.

#### Eastern Asian – Mediterranean disjunction in Theligonum

The Mediterranean species of *Theligonum* diverged from the three eastern Asian relatives at about 13.73 Mya (95% HPD: 6.19–23.24 Mya) in the middle Miocene. There are two potential scenarios for this western–eastern Eurasian disjunction: (1) vicariance followed by the rapid uplift of the QTP in the Miocene^55^ and/or enhanced aridity in the interior of Eurasia since 14 Mya^56,57^, and (2) long-distance dispersal.

The first scenario has been postulated as the major factor that shaped this disjunct pattern^19,58^ and geological data suggest that the rapid uplift of QTP (Fig. 3) occurred about 20 Mya^25,55,59,60^, although the details of this process remain controversial^59,61,62^. This event not only dramatically changed the topography of Asia but also Asian climate. The aridification of interior Asia is believed to begin about 22–25 Mya^63^ mainly due to the uplift of QTP^64–67^ although retreat of the Paratethys Sea also contributed significantly to desertification of Central Asia^68,69^. Recent magneto-stratigraphic data confirmed that the QTP uplift rather than sea retreat that occurred earlier in the middle Eocene, could be linked to the aridification of Central Asia at 25–20 Mya^70^.

Many plant and animal taxa have been reported to evolve in a response to the early Miocene QTP uplift during 14–24 Mya, e.g. *Cyananthus* ^71^, *Caragana* ^72^, *Ligularia–Cremanthodium–Parasenecio* complex^73^ and Chinese sisorid catfish^74^. Divergence time for *Theligonum* is dated back to about 14 Mya, close to the rapid uplift of QTP in the early to mid-Miocene, although there are many debates about the uplift processes of QTP at different times and different scales^10^. The QTP uplifted initially in the south-central Tibet very early (around 40 Mya) then uplifted around this part since 20 Mya, especially in the eastern, southern and northern regions^9^. Fossils contain a large amount of paleoenvironmental information and can be used as an efficient proxy for paleoelevation^10^. Based on geological and fossil evidences, the majority of QTP has reached the altitude of 2,000 m at ca. 15 Mya^25,62,75^. The Mediterranean species *T*. *cynocrambe* occurs at lower elevation (600–900 m), while the other three species are survived at 2500–2800 m in eastern Asia. The altitudinal distribution probably suggested a hypothesis that the uplift of the QTP above ca. 3000 m in the middle Miocene together with the aridification in Central Asia formed a barrier to gene exchange between eastern Asia and the Mediterranean area. In turn, our result suggests that the QTP has likely been reached nearly 3000 m in the middle Miocene.

Other explanations (long-distance dispersal and break–up of the migration pathway north of the QTP) are unlikely. The long-distance dispersal is not supported by seed morphology and dispersal mechanism. The nut-like fruits of *Theligonum* contain a very thin mesocarp^52,76^, and therefore can be dispersed only over short distances by small mammals^76–78^. Similarly, fruits of *T*. *cynocrambe* can be distributed only over short distance by ants consuming its mucilaginous seeds^39,52^. Discontinued by Pliocene climate fluctuations migration through north of the QTP has been proposed as an alternative explanation for more recent Asia and Europe disjunction^79^. However, the divergence time (13.73 Mya) is far older than the Pliocene. In addition, *Theligonum* is a relict of the Tethyan flora and the basal taxon of the temperate herbaceous group (*Theligonum*–*Kelloggia*–Rubieae) within the predominantly tropical and subtropical family Rubiaceae. Therefore an area north of the QTP may have been too cold for an ancestor of *Theligonum* to survive.

#### QTP – western North American disjunction in Kelloggia

The divergence time between eastern Asian *Kelloggia chinensis* and western North American *K*. *galioides* is estimated to be 18.52 Mya, close to that of *Theligonum*. This age estimate is not fully consistent with the Madrean–Tethyan hypothesis, which predicted earlier divergence of the intercontinental disjuncts^27^ (Fig. 3). Fossil records are very few for herbaceous taxa in Rubiaceae and no fossils are found in *Kelloggia*. Only two reliable pollen fossils reported from its closely related tribe Rubieae in the Miocene^80^ and provided very limited insights for the biogeography of *Kelloggia*. However, both paleontological and geological evidences suggested that the NALB which connected North America and Europe via southern Greenland and Scotland, and probably some island chains, served as the migration route for tropical, subtropical and temperate elements from the early to middle Cenozoic^2,4,81^. Nie *et al*.^40^ rejected the NALB pathway because the divergence time of *Kelloggia* in their analysis was too recent (5.42 ± 2.32 Mya). The present dating analysis based on a larger number of gene regions and credible fossil records, update the divergence time to be 18.52 Mya (95% HDP: 7.13–30.07 Mya), which is within the believed upper limit of 15 Mya^18,82,83^. Many thermophilic plants and a few animals are believed to have crossed the Atlantic within the past 15 Mya, including Bromeliaceae, Melastomataceae and Rapateaceae^84,85^. The BLB served another possible Cenozoic land connection between Eurasia and North America that contributed mainly to intercontinental temperate taxa exchange until about 3.5 Mya^2–4,86,87^.

According to our estimated divergence time, both the BLB and NALB could have acted as possible migration pathways for *Kelloggia*. Although, another hypothesis of long-distance dispersal (LDD) via birds is potentially possible in *Kelloggia* because its fruits possess hooked bristles. A similar situation is found in another disjunct genus *Osmorhiza* with hooked appendages on fruits^88^. The modern limited geographic range in each continent may be due to the limited availability of habitats in western North America and in the Hengduan Mountains. If we accept that *Kelloggia* originated within tropical Rubiaceae in the Tethyan region, and considering the fact that many close genera relatives occur in the extant Mediterranean region, the NALB hypothesis appears to be the most likely migration route. *Kelloggia*, as one of the Tethyan elements, might have expanded to North America via the NALB in the Oligocene to early Miocene (Fig. 3). Similar to *Theligonum*, the rapid uplift of QTP and aridification of Central Asia in early Miocene might cause its disjunction, and the climate cooling could be responsible for its modern restricted distribution.

Although the genus *Kelloggia* appears to have originated in the late Eocene to early Oligocene, modern species of *Kelloggia*, like *Theligonum*, arose much later (in Pliocene). Given the highest extinction frequency of Cenozoic relict taxa in Europe^4,16,18^, extinction of *Kelloggia* in Europe might occur during the climate change, and that could explain the results in our ancestor area reconstruction. The long branches between stem and crown of clades from each continent (clades 5 and 6 in Fig. 2) also suggest that extinction might be common in both genera.

## Conclusion

Our dating analysis and ancestral area reconstructions results suggest that both *Kelloggia* and *Theligonum* are Tethyan flora relicts, and the distribution area of their ancestors might have been warm tropical to subtropical environments along the Tethys coast in Eurasia. NALB appears to be the most likely pathway of expansion of *Kelloggia* to North America, though the LDD cannot be ruled out. A similar divergence time in *Kelloggia* and *Theligonum* (18.52 and 13.73 Mya) might reflect common historical events in their evolutionary history. The uplift of QTP in the early Miocene and aridification in Central Asia probably triggered separation of eastern and western parts of the ranges in both *Kelloggia* and *Theligonum*. Climate cooling since mid-Miocene in Northern Hemisphere caused range shifts in these two taxa to the modern distribution area, where *Theligonum* survived in moist micro-habitats of southwestern Eurasia while *Kelloggia* got extinct like many other Cenozoic relict taxa in this region^18^.

## Methods

### Taxon sampling

All species from both *Theligonum* and *Kelloggia* were included in this study. We newly sequenced three eastern Asian species of *Theligonum* (11 accessions), one species (*T*. *cynocrambe*) from Turkey (1 accession), and the *Kelloggia* species (5 accessions). Voucher information and accession numbers of newly sequenced taxa are provided in the Appendix S1. We also included 3 accessions of *Theligonum* and 5 accessions of *Kelloggia* from GenBank to cover the whole geographic range of both disjunct regions (Appendix S2). Close relatives of *Theligonum* and *Kelloggia* such as Rubieae, Putorieae, and Paederieae^45,51,89^, and the more distantly related *Gelsemium* ^90,91^ were included as outgroups in our phylogenetic analyses (Appendix S2).

### DNA extraction, amplification, and sequencing

Total genomic DNA was isolated from silica gel–dried leaf material using a Universal Genomic DNA Extraction Kit (Takara, Dalian, China). Five chloroplasts (the *rbc*L gene; the *rps*16 intron; the *trn*T–F region; the *atp*B–*rbc*L and *psb*A–*trn*H intergenic spacers) were selected for phylogenetic inference. For *trn*T*–*F regions, primers A (or A1) and D, as well as c and f as in Taberlet *et al*.^92^ and Bremer *et al*.^93^ were used with the internal. For *rbcL*, primers Z1 and 3′9^95^ were used. The *atp*B-*rbc*L and *psb*A-*trn*H spacers were amplified and sequenced using the primers as described by Manen *et al*.^96^ and Sang *et al*.^97^, respectively. The *rps*16 intron was amplified and sequenced with primers F and 2 R^98,99^. All polymerase chain reactions (PCRs) were run in a PTC–100 thermocycler (MJ Research, Ramsey, MN, USA). PCR products were purified using an agarose gel DNA purification kit (Takara, Shiga, Japan), following the manufacturer’s instructions. Sequencing was performed with BigDye Terminator 3.1 (Applied Biosystems, Foster City, CA, USA) on an ABI PRISM 3730 Sequencer using the same primers as employed for the PCR amplifications. All sequences were analyzed and assembled with Sequencher ver.4.14 (Gene Code, Ann Arbor, MI, USA).

### Sequence Alignment and Phylogenetic Analyses

DNA Baser v.3 (http://www.DnaBaser.com) was used to evaluate the chromatograms for base confirmation and to edit contiguous sequences. Multiple–sequence alignment was performed by MAFFT v.6^100^, using the default alignment parameters followed by manual adjustment in Se–Al v2.0a11 (http://tree.bio.ed.ac.uk/software/seal/), and gaps were treated as missing data.

DNA molecular phylogenies were reconstructed using maximum parsimony (MP), Bayesian inference (BI) and Maximum likelihood (ML). The parsimony analyses were conducted under the option heuristic search with 10 random stepwise additions and tree–bisection–reconnection (TBR) branch swapping with PAUP* version 4.0 b10^101^. Zero–length branches were collapsed and gaps were treated as missing data. Subsequently, parsimony bootstrap (BP) analyses^102^ with 1000 replicates were performed under the option fast and stepwise addition to evaluate the robustness of the MP trees.

Maximum likelihood (ML) analyses were run in RAxMLGUI 1.3^103^, followed by 1000 replicates of thorough Bootstrap (BS). Bayesian inference of likelihood was implemented by using with MrBayes version 3.1.2^104^. The best-fit models with parameters of nucleotide substitution for the individual data partitions were determined with jModeltest 2.1.3^105^ using the Akaike Information Criterion. For BI analyses, we ran two independent analyses consisting of four Markov chains sampled every 1000 generations for 10 million generations. After discarding the first 2 million generations as burn–in, the remaining trees from both analyses were pooled for a consensus tree. The proportions of bifurcations found in this consensus tree are given as Bayesian posterior probabilities (PP).

### Divergence Time Estimates and Fossil Calibration

Estimation of divergence times was performed in a Bayesian framework with BEAST version 1.7.5^106^ using the XSEDE package available online through the CIPRES Science Gateway 3.3^107^. After assessing the sequences generated and those available from GenBank, we chose to use the combined *rbcL*, *rps16*, *trnT-F*, and *atpB-rbcL* data to estimate the divergence time of *Theligonum* and *Kelloggia*. PsbA-trnH was not used due to its high sequences variation on the family level. With our focus on the divergence time of *Theligonum* and *Kelloggia*, and with the consideration of minimizing the influence from topological uncertainties in our analyses on dating of the phylogeny, we excluded some *Theligonum* and *Kelloggia* taxa. To allow multiple fossil calibrations in a broader phylogenetic framework of Rubiaceae, sequences of 68 additional taxa were obtained from GenBank (see Appendix S2). Furthermore, *Gelsemium sempervirens* (L.) J.St.-Hil. From Gelsemiaceae was selected as the remote outgroup in our dating analysis.

The input files were created using BEAUti 1.7.5, in which three partitions were specified. The best performing evolutionary model for each molecular marker was identified using jModelTest 2.1.1^105^. For the distribution of divergence times, a pure birth branching process (Yule model) was chosen as a prior. We ran two independent Markov chains, each for 100,000,000 generations, initiated with a random starting tree, and sampled every 10,000 generations. From collected samples, 15% were eliminated (treated as burn–in). All log and tree files from independent runs were combined using LogCombiner 1.7.5^106^. The results were summarized using the maximum clade credibility (MCC) tree option in TreeAnnotator 1.7.5^106^. Tracer 1.5 was used to check for convergence between the runs^106^. The tree was visualized using FigTree 1.4^108^ and the means and 95% higher posterior densities (HPD) could be obtained from it. The 95% HPD represents the shortest interval that contains 95% of the sampled values from the posterior^106^.

Four fossils from Rubiaceae (two fruits and two pollens) were selected as calibration points in our analyses, which have been widely used to estimate divergence times in various groups in the family^40,90,91,109–111^. The fruit fossil of *Cephalanthus* from the late Eocene to the Pliocene (see more in Antonelli *et al*.^90^), considered as the most convincing Rubiaceae fossils. The oldest fossil of *Cephalanthus* was found from Kireevski in western Siberia in the late Eocene^112,113^ and is used here to place a normal constraint of the stem age of *Cephalanthus* as 33.9 ± 1.0 Mya. Another well–preserved fruit fossil of a head–shaped infructescence was described as a new species, *Morinda chinensis* Shi, Liu & Jin, from the Changchang Formation in Hainan of China^114^. Because *Morinda* is paraphyletic in tribe Morideae^115^ and the phylogenetic position of this fossil species is unclear, we took a conservative approach and used this fossil to calibrate the crown age of the tribe with the prior set to 44.5 ± 3.0 Mya, which falls into the fossil age estimated from the late early Eocene to the early late Eocene^114^.

Most of the reported Rubiaceae fossils are dispersed pollen grains of a common tricolporate type. However, we used only the two most reliable pollen fossils in our analyses. The oldest pollen fossils of *Faramea* from the late Eocene (34–40 Mya) in Panama to the Pliocene in Veracruz, Mexico^80^, which are characterized by the orientation of the bacula at the apertures (two– to four–porate) and the size and the shape of the pollen^91,116^. Thus, the *Faramea* stem node was constrained at 37 ± 1.0 Mya. Two pollen fossils of *Scyphiphora* were reported at 16 Mya from Japan and at 23 Mya from the Marshall Islands in the northern Pacific Ocean^117,118^. *Scyphiphora* is the only extant genus of Rubiaceae that inhabits mangrove vegetation, and its pollen character is unique in the family, with distinct pores having a protruding papilla–like rim^91^. We therefore used 23 ± 1.0 Mya as a normal prior for the *Scyphiphora* node.

For rooting the tree, we followed Antonelli *et al*.^90^ to set the stem Rubiaceae age as 78 ± 1.0 Mya based on the crown age estimate of Gentianales^119^.

### Biogeographic analyses

Four biogeographical areas of endemism were delimited according to the main geographic distribution of *Theligonum*, *Kelloggia*, and its close relatives (Fig. 3): A = eastern Asia; B = the Mediterranean (including western Asia and north Africa); C = North America; D = Africa, but excluding north Africa.

Parsimony–based statistical dispersal–vicariance analysis (S–DIVA), accounting for uncertainty of both phylogenetic and ancestral area reconstructions^120–123^ was performed by RASP 2.0b^120,121^, and likelihood–based analyses under the dispersal–extinction–cladogenesis (DEC) model was performed with Lagrange 2.0.1^124,125^ to reconstruct ancestral areas at internal nodes. We conducted analyses with maximum alternative scenarios at each node set to 3 and 2 to examine the effect of constraints on reconstructed ancestral distributions^126^.

For the S–DIVA analyses, 8000 input trees were selected by resampling from the post–burn–in sample of the BEAST analysis at lower frequency using Log Combiner. Relative frequencies of ancestral areas reconstructed for each node were recorded and plotted onto the MCC tree from the BEAST analysis. The Lagrange online configurator (http://www.reelab.net/lagrange/configurator/index) was used to create input files. The MCC tree from the BEAST analysis was used as input trees.

## Electronic supplementary material

Supplementary Appendix
