# Supplementary material for: Role of the Qinghai-Tibetan Plateau uplift in the Northern Hemisphere disjunction: evidence from two herbaceous genera of Rubiaceae
Source: Sci Rep. 2017 Oct 17;7:13411. doi: 10.1038/s41598-017-13543-5 (PMC5645396; doi:10.1038/s41598-017-13543-5)
Supplement: Supplementary file 1 — Supplementary Appendix [file 41598_2017_13543_MOESM1_ESM.doc]

**ADDITIONAL INFORMATION**

**Role of the Qinghai–Tibetan Plateau uplift** **in the Northern Hemisphere disjunction: evidence from two herbaceous genera of Rubiaceae**

Tao Deng^1,3^, Jian-Wen Zhang^1,3^, Ying Meng^2^, Sergei Volis^1^, Hang Sun^1^,* , and Ze–Long Nie^2^,*

^1^Key Laboratory for Plant diversity and Biogeography of East Asia, Kunming Institute of Botany, Chinese Academy of Sciences, Kunming, Yunnan 650201, China. ^2^Key Laboratory of Plant Resources Conservation and Utilization, College of Biology and Environmental Sciences, Jishou University, Jishou, Hunan 416000, China. ^3^These authors contributed equally to the work.

* **Author for Correspondence:**

Hang Sun

[sunhang@mail.kib.ac.cn](mailto:sunhang@mail.kib.ac.cn), Fax: +86-871-65215002

Ze–Long Nie

[zelongnie@163.com](mailto:zelongnie@163.com)

**Running header:** Role of the QTP uplift in plant disjunction in two genera of Rubiaceae

**Supplementary Appendix S1** **Voucher information and accession numbers of newly investigated taxa in the phylogenetic analyses.**

| *Taxon* | Collection locality | Voucher | *rbc*L | *trn*T-F | *rps*16 | *atp*B*-rbc*L | *psb*A*-trn*H |
| --- | --- | --- | --- | --- | --- | --- | --- |
| *Kelloggia chinensis* | China, Sichuan | *Nie et al.* 2661 (KUN) | MF780747 | MF780797 | MF780765 | MF780782 | MF780735 |
| *Kelloggia chinensis* | China, Xizang | *Nie et al.* 3197 (KUN) | MF780748 | MF780798 | MF780766 | MF780783 | MF780736 |
| *Kelloggia chinensis* | China, Sichuan | dt37 (KUN) | MF780749 | MF780799 | MF780767 | MF780784 | MF780737 |
| *Kelloggia chinensis* | China, Yunnan | dt38 (KUN) | MF780750 | ─ | MF780768 | MF780785 | MF780738 |
| *Kelloggia chinensis* | China, Yunnan | *Nie et al.* 1477 (KUN) | MF780751 | MF780800 | MF780769 | ─ | MF780739 |
| *Rubia chinensis* | China, Guizhou | *Nie et al.* 2125 (KUN) | MF780752 | MF780801 | ─ | MF780786 | MF780740 |
| *Theligonum cynocrambe* | Turkey | *Sun et al.* 18240 (KUN) | MF780753 | ─ | MF780770 | MF780787 | MF780741 |
| *Theligonum formosanum* | China, Taiwan | *Nie et al.* 756 (KUN) | MF780754 | MF780802 | MF780771 | MF780788 | MF780742 |
| *Theligonum formosanum* | China, Taiwan | *Sun et al. 11998* (KUN) | MF780755 | ─ | MF780772 | MF780789 | ─ |
| *Theligonum formosanum* | China, Taiwan | *Sun et al. 12133* (KUN) | MF780756 | ─ | MF780773 | MF780790 | ─ |
| *Theligonum formosanum* | China, Taiwan | *Sun et al.* *12161* (KUN) | MF780757 | ─ | MF780774 | MF780791 | ─ |
| *Theligonum japonicum* | Japan | *Nie et al.* 775 (KUN) | MF780758 | ─ | MF780775 | MF780792 | ─ |
| *Theligonum japonicum* | Japan | *Soejima,* 1117 (KUN) | MF780759 | MF780803 | MF780776 | ─ | ─ |
| *Theligonum macranthum* | China, Anhui | *Deng & Zhang,* 003 (KUN) | MF780760 | MF780804 | MF780777 | MF780793 | MF780743 |
| *Theligonum macranthum* | China, Hubei | *Nie et al.* 717 (KUN) | MF780761 | MF780805 | MF780778 | MF780794 | ─ |
| *Theligonum macranthum* | China, Hubei | *Zhang et al.* 476 (KUN) | MF780762 | MF780806 | MF780779 | ─ | MF780744 |
| *Theligonum macranthum* | China, Hunan | *Zhang et al.* 560 (KUN) | MF780763 | MF780807 | MF780780 | MF780795 | MF780745 |
| *Theligonum macranthum* | China, Zhejiang | *Deng & Zhang,* 052 (KUN) | MF780748 | MF780798 | MF780766 | MF780783 | MF780736 |

**Supplementary Appendix S2 Sequences obtained from GenBank and used in the pphylogenetic and divergence time analyses**.

| *Taxon* | *rbc*L | *trn*T-F | *rps*16 | *atp*B*-rbc*L | *psb*A*-trn*H |
| --- | --- | --- | --- | --- | --- |
| *Aitchisonia rosea* | DQ662172[^1^](#_ENREF_1) | DQ662134[^1^](#_ENREF_1) | DQ662195[^1^](#_ENREF_1) | ─ | ─ |
| *Alberta minor* | EU817410[^2^](#_ENREF_2) | EU817452[^2^](#_ENREF_2) | EF205637[^3^](#_ENREF_3) | DQ131699* | ─ |
| *Anthospermum herbaceum* | X83623[^4^](#_ENREF_4) | EU145544[^5^](#_ENREF_5) | EU145496[^5^](#_ENREF_5) | AJ234028[^6^](#_ENREF_6) | ─ |
| *Appuniaguatemalensis* | AJ288593[^6^](#_ENREF_6) | AM945332[^7^](#_ENREF_7) | ─ | AJ234009[^6^](#_ENREF_6) | ─ |
| *Asperulapurpurea* | FR865121[^8^](#_ENREF_8) | GU357130[^9^](#_ENREF_9) | ─ | X81686[^10^](#_ENREF_10) | FR865075[^8^](#_ENREF_8) |
| *Calycophyllumcandidissimum* | X83627[^4^](#_ENREF_4) | DQ359164* | AF004030[^11^](#_ENREF_11) | DQ131708* | ─ |
| *Captaincookiamargaretae* | EU817415[^2^](#_ENREF_2) | EU817456[^2^](#_ENREF_2) | EU817436[^2^](#_ENREF_2) | ─ | ─ |
| *Catesbaea spinosa* | X83628[^4^](#_ENREF_4) | AF152706[^12^](#_ENREF_12) | AF004032[^11^](#_ENREF_11) | ─ | ─ |
| *Cephalanthus occidentalis* | X83629[^4^](#_ENREF_4) | AF152692[^12^](#_ENREF_12) | AF004033[^11^](#_ENREF_11) | DQ131710* | ─ |
| *Choulettiareboudiana* | DQ662173[^1^](#_ENREF_1) | DQ662137[^1^](#_ENREF_1) | DQ662196[^1^](#_ENREF_1) | ─ | ─ |
| *Cinchona pubescens* | X83630[^4^](#_ENREF_4) | AY538451[^13^](#_ENREF_13) | AF004035[^11^](#_ENREF_11) | AJ233990[^6^](#_ENREF_6) | ─ |
| *Coccocypselum condalia* | AM117217[^14^](#_ENREF_14) | EU145547[^5^](#_ENREF_5) | EU145499[^5^](#_ENREF_5) | EU145324[^5^](#_ENREF_5) | ─ |
| *Condaminea corymbosa* | Y18713[^15^](#_ENREF_15) | AF102406* | AF004039[^11^](#_ENREF_11) | ─ | ─ |
| *Coussarea hydrangeifolia* | EU145460[^5^](#_ENREF_5) | EU145549[^5^](#_ENREF_5) | EU145501[^5^](#_ENREF_5) | EU145326[^5^](#_ENREF_5) | ─ |
| *Cremaspora triflora* | Z68856[^16^](#_ENREF_16) | AF201040[^17^](#_ENREF_17) | AF200990[^17^](#_ENREF_17) | DQ131718* | ─ |
| *Cruciata glabra* | X81097[^18^](#_ENREF_18) | GU357109[^9^](#_ENREF_9) | ─ | X76470[^18^](#_ENREF_18) | ─ |
| *Cruckshanksia hymenodon* | AJ288599[^6^](#_ENREF_6) | EU145550[^5^](#_ENREF_5) | EU145502[^5^](#_ENREF_5) | AJ234004[^6^](#_ENREF_6) | ─ |
| *Damnacanthus macrophyllus* | AM945285[^7^](#_ENREF_7) | AM945336[^7^](#_ENREF_7) | AM945308[^7^](#_ENREF_7) | AM945222[^7^](#_ENREF_7) | ─ |
| *Danaisxanthorrhoea* | Z68794[^16^](#_ENREF_16) | AM409329[^19^](#_ENREF_19) | AM117297[^14^](#_ENREF_14) | AJ234019[^6^](#_ENREF_6) | ─ |
| *Declieuxiafruticosa* | AJ002177[^20^](#_ENREF_20) | EU145552[^5^](#_ENREF_5) | EU145503[^5^](#_ENREF_5) | DQ131721* | ─ |
| *Didymaeaalsinoides* | Z68795[^16^](#_ENREF_16) | EU145570[^5^](#_ENREF_5) | ─ | AJ234036[^6^](#_ENREF_6) | ─ |
| *Diplosporapolysperma* | AJ286703[^21^](#_ENREF_21) | EU145538[^5^](#_ENREF_5) | AM117301[^14^](#_ENREF_14) | ─ | ─ |
| *Doriceratrilocularis* | EU817417[^2^](#_ENREF_2) | EU817457[^2^](#_ENREF_2) | EU817437[^2^](#_ENREF_2) | ─ | ─ |
| *Emmenopterys henryi* | Y18715[^15^](#_ENREF_15) | AF152637[^12^](#_ENREF_12) | AF242941* | DQ131728* | ─ |
| *Exostema spinosum* | AY205350[^22^](#_ENREF_22) | AY763830[^23^](#_ENREF_23) | AF242947* | ─ | ─ |
| *Faramea multiflora* | Z68796[^16^](#_ENREF_16) | AF102422* | AF004048[^11^](#_ENREF_11) | EU145328[^5^](#_ENREF_5) | ─ |
| *Ferdinandusaspeciosa* | Y18713[^15^](#_ENREF_15) | EU145534[^5^](#_ENREF_5) | AM117304[^14^](#_ENREF_14) | DQ131735* | ─ |
| *Gailloniaolivieri* | DQ662176[^1^](#_ENREF_1) | DQ662142[^1^](#_ENREF_1) | DQ662199[^1^](#_ENREF_1) | ─ | ─ |
| *Galium album* | X81090[^18^](#_ENREF_18) | GU357146[^9^](#_ENREF_9) | AF004050[^11^](#_ENREF_11) | X76459[^18^](#_ENREF_18) | ─ |
| *Galium elongatum* | X81098[^18^](#_ENREF_18) | GU357122[^9^](#_ENREF_9) | ─ | X76461[^18^](#_ENREF_18) | ─ |
| *Galium scabrum* | X81105[^18^](#_ENREF_18) | GU357101[^9^](#_ENREF_9) | ─ | X76462[^18^](#_ENREF_18) | ─ |
| *Gelsemium sempervirens* | L14397[^24^](#_ENREF_24) | AM295096[^25^](#_ENREF_25) | DQ660581[^26^](#_ENREF_26) | AJ233985[^6^](#_ENREF_6) | ─ |
| *Guettarda uruguensis* | X83638[^4^](#_ENREF_4) | EU145533[^5^](#_ENREF_5) | EU145489[^5^](#_ENREF_5) | DQ131739* | ─ |
| *Gynochthodes coriacea* | AJ288603[^6^](#_ENREF_6) | AJ847407[^27^](#_ENREF_27) | AM117311[^14^](#_ENREF_14) | AM945219[^7^](#_ENREF_7) | ─ |
| *Hillia triflora* | X83642[^4^](#_ENREF_4) | AM117362[^14^](#_ENREF_14) | AM117315[^14^](#_ENREF_14) | AJ233993[^6^](#_ENREF_6) | ─ |
| *Isertia coccinea* | AY538489[^13^](#_ENREF_13) | AY538455[^13^](#_ENREF_13) | AY538430[^13^](#_ENREF_13) | ─ | ─ |
| *Ixoracoccinea* | X83646[^4^](#_ENREF_4) | EU817464[^2^](#_ENREF_2) | EF205641[^3^](#_ENREF_3) | AM412400* | ─ |
| *Jaubertia aucheri* | DQ662178[^1^](#_ENREF_1) | DQ662145[^1^](#_ENREF_1) | DQ662202[^1^](#_ENREF_1) | FJ695383[^28^](#_ENREF_28) | ─ |
| *Kelloggia chinensis* | AY570775[^29^](#_ENREF_29) | ─ | AY570769[^29^](#_ENREF_29) | AY570764[^29^](#_ENREF_29) | ─ |
| *Kelloggia chinensis* | AY570776[^29^](#_ENREF_29) | ─ | AY570770[^29^](#_ENREF_29) | AY570765[^29^](#_ENREF_29) | ─ |
| *Kelloggia galioides* | AY570777[^29^](#_ENREF_29) | ─ | AY570772[^29^](#_ENREF_29) | AY570766[^29^](#_ENREF_29) | ─ |
| *Kelloggia galioides* | AY570778[^29^](#_ENREF_29) | ─ | AY570773[^29^](#_ENREF_29) | AY570767[^29^](#_ENREF_29) | ─ |
| *Kelloggia galioides* | DQ662179[^1^](#_ENREF_1) | DQ662146[^1^](#_ENREF_1) | DQ662203[^1^](#_ENREF_1) | ─ | ─ |
| *Kraussia floribunda* | Z68858[^16^](#_ENREF_16) | AM117368[^14^](#_ENREF_14) | AM117325[^14^](#_ENREF_14) | DQ131746* | ─ |
| *Leptodermispotanini* | DQ662180[^1^](#_ENREF_1) | DQ662148[^1^](#_ENREF_1) | DQ662204[^1^](#_ENREF_1) | ─ | ─ |
| *Luculiagrandifolia* | X83648[^4^](#_ENREF_4) | DQ662149[^1^](#_ENREF_1) | DQ662205[^1^](#_ENREF_1) | AJ233986[^6^](#_ENREF_6) | ─ |
| *Mitchella repens* | JX412470[^30^](#_ENREF_30) | JX412387[^30^](#_ENREF_30) | JX412449[^30^](#_ENREF_30) | JX412408[^30^](#_ENREF_30) | ─ |
| *Morinda citrifolia* | AJ318448[^31^](#_ENREF_31) | AF152616[^12^](#_ENREF_12) | AJ320078[^31^](#_ENREF_31) | ─ | ─ |
| *Mussaendaerythrophylla* | X83652[^4^](#_ENREF_4) | EU145535[^5^](#_ENREF_5) | EU145493[^5^](#_ENREF_5) | DQ131754* | ─ |
| *Nauclea orientalis* | AY538501[^13^](#_ENREF_13) | AJ346958[^32^](#_ENREF_32) | AJ320080[^31^](#_ENREF_31) | EU145320[^5^](#_ENREF_5) | ─ |
| *Ophiorrhiza mungos* | FJ209082* | DQ662151[^1^](#_ENREF_1) | AF004064[^11^](#_ENREF_11) | FJ226541* | ─ |
| *Pentaslanceolata* | L13931* | AM266963[^33^](#_ENREF_33) | AM266875[^33^](#_ENREF_33) | AB247149[^34^](#_ENREF_34) | ─ |
| *Plocamaaucheri* | ─ | DQ662145[^1^](#_ENREF_1) | DQ662202[^1^](#_ENREF_1) | FJ695383[^28^](#_ENREF_28) | ─ |
| *Plocamapendula* | Z68816[^35^](#_ENREF_35) | DQ662162[^1^](#_ENREF_1) | AF004071[^11^](#_ENREF_11) | AJ234035[^6^](#_ENREF_6) | ─ |
| *Pseudogailloniahymenostephana* | DQ662190[^1^](#_ENREF_1) | DQ662163[^1^](#_ENREF_1) | DQ662215[^1^](#_ENREF_1) | -- | ─ |
| *Pterogailloniacalycoptera* | DQ662191[^1^](#_ENREF_1) | DQ662166[^1^](#_ENREF_1) | DQ662216[^1^](#_ENREF_1) | -- | ─ |
| *Putoriacalabrica* | AJ288620[^6^](#_ENREF_6) | DQ662166[^1^](#_ENREF_1) | AF004072[^11^](#_ENREF_11) | X81672[^18^](#_ENREF_18) | ─ |
| *Rondeletiaodorata* | Y11857[^36^](#_ENREF_36) | AF152741[^12^](#_ENREF_12) | EU145490[^5^](#_ENREF_5) | EU145321[^5^](#_ENREF_5) | ─ |
| *Rubiatinctorum* | X83666[^4^](#_ENREF_4) | FJ695421[^28^](#_ENREF_28) | ─ | X76465[^18^](#_ENREF_18) | EU531723* |
| *Sabicea diversifolia* | EU145459[^5^](#_ENREF_5) | AJ847396[^27^](#_ENREF_27) | EU145494[^5^](#_ENREF_5) | DQ131781* | ─ |
| *Saprosma foetens* | DQ662193[^1^](#_ENREF_1) | DQ662168[^1^](#_ENREF_1) | DQ662218[^1^](#_ENREF_1) | FJ695386[^28^](#_ENREF_28) | ─ |
| *Scyphiphora hydrophyllace* | EU817432[^2^](#_ENREF_2) | EU817475[^2^](#_ENREF_2) | DQ923045* | ─ | ─ |
| *Serissa foetida* | Z68822* | AF152618[^12^](#_ENREF_12) | AF004081[^11^](#_ENREF_11) | AJ234034[^6^](#_ENREF_6) | ─ |
| *Sherardiaarvensis* | HM850351[^37^](#_ENREF_37) | EU145571[^5^](#_ENREF_5) | AF004082[^11^](#_ENREF_11) | X76458[^18^](#_ENREF_18) | HE966811[^38^](#_ENREF_38) |
| *Spermadictyonsuaveolens* | Z68824* | DQ662171[^1^](#_ENREF_1) | DQ662219[^1^](#_ENREF_1) | ─ | ─ |
| *Theligonum cynocrambe* | X83668[^4^](#_ENREF_4) | AF152621[^12^](#_ENREF_12) | AF004087[^11^](#_ENREF_11) | X81680[^18^](#_ENREF_18) | ─ |
| *Theligonum cynocrambe* | FJ695248[^28^](#_ENREF_28) | FJ695426[^28^](#_ENREF_28) | ─ | FJ695393[^28^](#_ENREF_28) | ─ |
| *Theligonum cynocrambe* | FJ695249[^28^](#_ENREF_28) | FJ695427[^28^](#_ENREF_28) | ─ | ─ | ─ |
| *Uncariarhynchophylla* | X83669[^4^](#_ENREF_4) | AJ346959[^32^](#_ENREF_32) | AB178637[^39^](#_ENREF_39) | ─ | ─ |
| *Valantiahispida* | FJ695251[^28^](#_ENREF_28) | AM117385[^14^](#_ENREF_14) | AF004090[^11^](#_ENREF_11) | FJ695395[^28^](#_ENREF_28) | ─ |
| *Versteegiacauliflora* | EU817433[^2^](#_ENREF_2) | EU817476[^2^](#_ENREF_2) | EU817451[^2^](#_ENREF_2) | ─ | ─ |
| *Xanthophytum borneense* | EU145466[^5^](#_ENREF_5) | EU145567[^5^](#_ENREF_5) | EU145513[^5^](#_ENREF_5) | EU145335[^5^](#_ENREF_5) | ─ |

Reference

1 Backlund, M., Bremer, B. & Thulin, M. Paraphyly of Paederieae, recognition of Putorieae and expansion of *Plocama* (Rubiaceae-Rubioideae). *Taxon* **56**, 315-328 (2007).

2 Mouly, A., Razafimandimbison, S. G., Florence, J., Jérémie, J. & Bremer, B. Paraphyly of *Ixora* and new tribal delimitation of Ixoreae (Rubiaceae): inference from combined chloroplast (rps16, rbcL, and trnT-F) sequence data. *Annals of the Missouri Botanical Garden* **96**, 146-160, doi:doi:10.3417/2006194 (2009).

3 Mouly, A., Razafimandimbison, S. G., Achille, F., Haevermans, T. & Bremer, B. Phylogenetic placement of *Rhopalobrachium fragrans* (Rubiaceae): Evidence from molecular (rps16 and trnT-F) and morphological data. *Systematic Botany* **32**, 872-882 (2007).

4 Bremer, B., K Andreasen & Olsson, D. Subfamilial and tribal relationships in the Rubiaceae based on rbcL sequence data. *Ann Mo Bot Gard* **82**, 383–397 (1995).

5 Rydin, C., Razafimandimbison, S. G. & Bremer, B. Rare and enigmatic genera (*Dunnia*, *Schizocolea*, *Colletoecema*), sisters to species-rich clades: phylogeny and aspects of conservation biology in the coffee family. *Molecular Phylogenetics and Evolution* **48**, 74-83, doi:10.1016/j.ympev.2008.04.006 (2008).

6 Bremer, B. & Manen, J. F. Phylogeny and classification of the subfamily Rubioideae (Rubiaceae). *Plant Systematics and Evolution* **225**, 43-72 (2000).

7 Razafimandimbison, S. G., Rydin, C. & Bremer, B. Evolution and trends in the Psychotrieae alliance (Rubiaceae) - A rarely reported evolutionary change of many-seeded carpels from one-seeded carpels. *Molecular Phylogenetics and Evolution* **48**, 207-223, doi:10.1016/j.ympev.2008.03.034 (2008).

8 De Mattia, F. *et al.* A multi-marker DNA barcoding approach to save time and resources in vegetation surveys. *Botanical Journal of the Linnean Society* **169**, 518-529, doi:10.1111/j.1095-8339.2012.01251.x (2012).

9 Soza, V. L. & Olmstead, R. G. Molecular systematics of tribe Rubieae (Rubiaceae): Evolution of major clades, development of leaf-like whorls, and biogeography. *Taxon* **59**, 755-771 (2010).

10 Natali, A., Manen, J.-F. & Ehrendorfer, F. Phylogeny of the Rubiaceae-Rubioideae, in particular the tribe Rubieae: evidence from a non-coding chloroplast DNA sequence. *Annals of the Missouri Botanical Garden* **82**, 428-439 (1995).

11 Andersson, L. & Rova, J. H. The rps16 intron and the phylogeny of the Rubioideae (Rubiaceae). *Plant Systematics and Evolution* **214**, 161-186 (1999).

12 Rova, J. H. E., Delprete, P. G., Andersson, L. & Albert, V. A. A trnL-F cpDNA sequence study of the Condamineeae-Rondeletieae-Sipaneeae complex with implications on the phylogeny of the Rubiaceae. *American Journal of Botany* **89**, 145-159, doi:DOI 10.3732/ajb.89.1.145 (2002).

13 Andersson, L. & Antonelli, A. Phylogeny of the tribe Cinchoneae (Rubiaceae), its position in Cinchonoideae, and description of a new genus, Ciliosemina. *Taxon* **54**, 17-28 (2005).

14 Bremer, B. & Eriksson, T. Time tree of Rubiaceae: phylogeny and dating the family, subfamilies, and tribes. *International Journal of Plant Sciences* **170**, 766-793, doi:doi:10.1086/599077 (2009).

15 Bremer, B. *et al.* More characters or more taxa for a robust phylogeny - case study from the coffee family (Rubiaceae). *Systematic Biology* **48**, 413-435 (1999).

16 Andreasen, K. & Bremer, B. Phylogeny of the subfamily Ixoroideae (Rubiaceae). *Opera Bot Belg* **7**, 119-138 (1996).

17 Persson, C. Phylogeny of Gardenieae (Rubiaceae) based on chloroplast DNA sequences from the rps16 intron and trnL(UAA)-F(GAA) intergenic spacer. *Nordic Journal of Botany* **20**, 257-269, doi:DOI 10.1111/j.1756-1051.2000.tb00742.x (2000).

18 Manen, J. F. & Natali, A. Comparison of the evolution of ribulose-1,5-biphosphate carboxylase (rbcL) and atpB-rbcL noncoding spacer sequences in a recent plant group, the tribe Rubieae (Rubiaceae). *Journal of Molecular Evolution* **41**, 920-927 (1995).

19 Khan, S. A., Razafimandimbison, S. G., Bremer, B. & Liede-Schumann, S. Sabiceeae and Virectarieae (Rubiaceae, Ixoroideae): one or two tribes? New tribal and generic circumscriptions of Sabiceeae and biogeography of Sabicea s.l. *Taxon* **57**, 7-23 (2008).

20 Nepokroeff, M., Bremer, B. & Sytsma, K. J. Reorganization of the genus Psychotria and tribe Psychotrieae (Rubiaceae) inferred from ITS and rbcL sequence data. *Systematic Botany* **24**, 5-27 (1999).

21 Andreasen, K. & Bremer, B. Combined phylogenetic analysis in the Rubiaceae-Ixoroideae: Morphology, nuclear and chloroplast DNA data. *American Journal of Botany* **87**, 1731-1748, doi:Doi 10.2307/2656750 (2000).

22 McDowell, T., Volovsek, M. & Manos, P. Biogeography of *Exostema* (Rubiaceae) in the Caribbean region in light of molecular phylogenetic analyses. *Systematic Botany* **28**, 431-441 (2003).

23 Motley, T. J., Wurdack, K. J. & Delprete, P. G. Molecular systematics of the Catesbaeeae-Chiococceae complex (Rubiaceae): Flower and fruit evolution and biogeographic implications. *American Journal of Botany* **92**, 316-329, doi:DOI 10.3732/ajb.92.2.316 (2005).

24 Olmstead, R. G., Bremer, B., Scott, K. M. & Palmer, J. D. A parsimony analysis of the Asteridae sensu lato based on rbcL sequences. *Annals of the Missouri Botanical Garden* **80**, 700-722 (1993).

25 Endress, M. E. *et al.* A phylogenetic analysis of alyxieae (Apocynaceae) based on rbcL, matK, trnL intron, trnL-F spacer sequences, and morphological characters. *Annals of the Missouri Botanical Garden* **94**, 1-35, doi:Doi 10.3417/0026-6493(2007)94[1:Apaoaa]2.0.Co;2 (2007).

26 Simoes, A. O., Livshultz, T., Conti, E. & Endress, M. E. Phylogeny and systematics of the Rauvolfioideae (Apocynaceae) based on molecular and morphological evidence. *Annals of the Missouri Botanical Garden* **94**, 268-297 (2007).

27 Alejandro, G. D., Razafimandimbison, S. G. & Liede-Schumann, S. Polyphyly of mussaenda inferred from its and trnT-F data and its implication for generic limits in mussaendeae (Rubiaceae). *American Journal of Botany* **92**, 544-557 (2005).

28 Rydin, C., Razafimandimbison, S. G., Khodabandeh, A. & Bremer, B. Evolutionary relationships in the Spermacoceae alliance (Rubiaceae) using information from six molecular loci: insights into systematic affinities of Neohymenopogon and Mouretia. *Taxon* **58**, 793-810 (2009).

29 Nie, Z.-L., Wen, J., Sun, H. & Bartholomew, B. Monophyly of *Kelloggia* Torrey ex Benth. (Rubiaceae) and evolution of its intercontinental disjunction between western North America and eastern Asia. *American Journal of Botany* **92**, 642-652, doi:10.3732/ajb.92.4.642 (2005).

30 Huang, W.-P. *et al.* Molecular phylogenetics and biogeography of the eastern Asian–eastern North American disjunct *Mitchella* and its close relative *Damnacanthus* (Rubiaceae, Mitchelleae). *Botanical Journal of the Linnean Society* **171**, 395-412, doi:10.1111/j.1095-8339.2012.01321.x (2013).

31 Novotny, V. *et al.* Low host specificity of herbivorous insects in a tropical forest. *Nature* **416**, 841-844, doi:DOI 10.1038/416841a (2002).

32 Razafimandimbison, S. G. & Bremer, B. Phylogeny and classification of Naucleeae s.l. (Rubiaceae) inferred from molecular (ITS, rBCL, and tRNT-F) and morphological data. *American Journal of Botany* **89**, 1027-1041, doi:10.3732/ajb.89.7.1027 (2002).

33 McNeill, J. & Turland, N. J. The systematics of Knoxieae (Rubiaceae)-molecular data and their taxonomic consequences (vol 56, pg 1051, 2007). *Taxon* **58**, 317-317 (2009).

34 Nakamura, K., Chung, S. W., Kokubugata, G., Denda, T. & Yokota, M. Phylogenetic systematics of the monotypic genus *Hayataella* (Rubiaceae) endemic to Taiwan. *Journal of Plant Research* **119**, 657-661, doi:10.1007/s10265-006-0017-4 (2006).

35 Bremer, B. Phylogenetic studies within Rubiaceae and relationships to other families based on molecular data. *Opera Bot Belg* **7**, 33-50 (1996).

36 Bremer, B. & Thulin, M. Collapse ofIsertieae, re-establishment ofMussaendeae, and a new genus of *Sabiceeae* (Rubiaceae); phylogenetic relationships based onrbcL data. *Plant Systematics and Evolution* **211**, 71-92, doi:10.1007/bf00984913 (1998).

37 Schaefer, H., Hardy, O. J., Silva, L., Barraclough, T. G. & Savolainen, V. Testing Darwin's naturalization hypothesis in the Azores. *Ecology Letters* **14**, 389-396, doi:Doi 10.1111/J.1461-0248.2011.01600.X (2011).

38 Bruni, I. *et al.* DNA barcoding as an effective tool in improving a digital plant identification system: A case study for the area of Mt. Valerio, Trieste (NE Italy). *Plos One* **7**, doi:ARTN e43256 10.1371/journal.pone.0043256 (2012).

39 Aoki, K., Suzuki, T. & Murakami, N. Intraspecific sequence variation of chloroplast DNA among the component species of evergreen broad-leaved forests in Japan. *Journal of Plant Research* **116**, 337-344, doi:10.1007/s10265-003-0103-9 (2003).
